# Residual soil nitrate content and profitability of five cropping systems in northwest Iowa

**DOI:** 10.1371/journal.pone.0171994

**Published:** 2017-03-01

**Authors:** Robert L. De Haan, Matthew A. Schuiteman, Ronald J. Vos

**Affiliations:** 1 Environmental Studies Department, Dordt College, Sioux Center, Iowa, United States of America; 2 AJS Farms, Sioux Center, Iowa, United States of America; 3 Agriculture Department, Dordt College, Sioux Center, Iowa, United States of America; USDA Agricultural Research Service, UNITED STATES

## Abstract

Many communities in the Midwestern United States obtain their drinking water from shallow alluvial wells that are vulnerable to contamination by NO_3_-N from the surrounding agricultural landscape. The objective of this research was to assess cropping systems with the potential to produce a reasonable return for farmers while simultaneously reducing the risk of NO_3_-N movement into these shallow aquifers. From 2009 to 2013 we conducted a field experiment in northwest Iowa in which we evaluated five cropping systems for residual (late fall) soil NO_3_-N content and profitability. Soil samples were taken annually from the top 30 cm of the soil profile in June and August, and from the top 180 cm in November (late fall). The November samples were divided into 30 cm increments for analysis. Average residual NO_3_-N content in the top 180 cm of the soil profile following the 2010 to 2013 cropping years was 134 kg ha^-1^ for continuous maize (*Zea mays* L.) with a cereal rye (*Secale cereale* L.) cover crop, 18 kg ha^-1^ for perennial grass, 60 kg ha^-1^ for a three year oat (*Avena sativa* L.)-alfalfa (*Medicago sativa* L.)-maize rotation, 85 kg ha^-1^ for a two year oat/red clover (*Trifolium pratense* L.)-maize rotation, and 90 kg ha^-1^ for a three year soybean (*Glycine max* (L.) Merr.)-winter wheat (*Triticum aestivum* L.)-maize rotation. However, residual NO_3_-N in the 90 to 180 cm increment of the soil profile was not significantly higher in the oat-alfalfa-maize cropping system than the perennial grass system. For 2010 to 2013, average profit ($ ha^-1^ yr^-1^) was 531 for continuous corn, 347 for soybean-winter wheat-maize, 264 for oat-alfalfa-maize, 140 for oat/red clover-maize, and -384 (loss) for perennial grass. Considering both residual soil NO_3_-N and profitability data, the oat-alfalfa-maize rotation performed the best in this setting. However, given current economic pressures widespread adoption is likely to require changes in public policy.

## Introduction

More than 150 community water supplies in Iowa obtain water from shallow wells located in alluvial aquifers that are highly susceptible to contamination [1]. These aquifers are typically overlain and/or surrounded by intensive crop production systems dominated by fields of maize and soybean, which tend to “leak” NO_3_-N even when best management practices are utilized [2,3]. In some situations, NO_3_-N derived from fertilizer may continue to move down through the soil profile and impact water quality for many years after application [4]. As a result, these wells are at risk of producing water with NO_3_-N concentrations exceeding the United States Environmental Protection Agency’s maximum contaminant level of 10 mg L^-1^ [5]. This situation is common throughout the Midwestern U.S [6]. In Nebraska, high concentrations of NO_3_-N in groundwater from agricultural areas were first documented in 1961, and have been of concern since then [7]. Nitrate-nitrogen concentrations in groundwater in rural Minnesota are also problematic in parts of the state and are being monitored [8].

The city of Sioux Center, located in northwest Iowa, obtains more than 50% of its drinking water from an alluvial aquifer along the West Branch of the Floyd River (43°08´ N; 96°11´ W; 407 m above sea level). The aquifer is accessed by 12 wells that are 9 to 13 m deep. These shallow wells, like those in many Midwestern communities, are strongly influenced by land management practices in the immediate vicinity and in the surrounding watershed. In recent years, several of these wells have yielded water with NO_3_-N concentrations greater than 10 mg L^-1^. The city blends water from its shallow wells with water from other sources to ensure the NO_3_-N concentration in the finished drinking water remains below 10 mg L^-1^.

Midwestern communities like Sioux Center, seeking to reduce NO_3_-N concentrations in water produced by their shallow alluvial wells, can often benefit by altering the vegetation grown in the capture zone surrounding the wells. If the municipality owns the land, it can enroll it in the Conservation Reserve Program–Wellhead Protection Program (CRP-WHP) and receive rental payments for it from the federal government [1,9]. Land enrolled in the program is typically seeded to a deep-rooted perennial grass and is not harvested. If the municipality doesn’t own all the land in the capture zone (a common occurrence), adjacent private landowners can be encouraged to enroll their land in this program, but are not obligated to do so. In Iowa, 2401 ha of land were enrolled in the CRP-WHP program as of 2014 (S1 File). This comprises a small percentage of the eligible land [1], indicating that many landowners are unaware of the program or do not see it as an attractive choice.

Rural communities and private landowners do have other options. Cropping systems utilizing plant species in addition to, or instead of, maize and soybean have the potential to reduce NO_3_-N losses to groundwater and to be profitable [10–13]. The identification of alternative cropping systems that reduce the likelihood of NO_3_-N leaching, while still generating adequate income for the landowner/operator, could facilitate changes in farming practices that improve water quality for many rural communities.

The objectives of this research were to assess cropping systems with the potential to: (1) produce a reasonable return for farmers and (2) simultaneously reduce residual soil NO_3_-N content, and therefore the risk of NO_3_-N movement into shallow municipal aquifers. We also wanted to share the results with those who could benefit from the information (agricultural producers, municipalities, the Natural Resources Conservation Service, the Iowa Department of Natural Resources, and the research community). We hypothesized that intentionally designed and carefully managed cropping systems would have soil NO_3_-N contents similar to those in perennial vegetation typical of Conservation Reserve Program plantings, but would generate additional income for the land owner/operator, and therefore be implemented more readily. We successfully evaluated five cropping systems for residual NO_3_-N content and profitability, and were able to identify several promising crop production strategies for use on land surrounding shallow alluvial wells.

## Materials and methods

### Site description

We conducted our experiment on a 16 ha site 4 km east of Sioux Center, Iowa (43°07´ N; 96°10´ W; 412 m above sea level). The site was about 300 m east of the West Branch of the Floyd River where 12 shallow alluvial wells utilized by the city of Sioux Center were located. The surrounding land area was intensively farmed; the main crops were rainfed maize and soybean. Soils at the site were Mollisols, with the predominant soil type being a Galva silty clay loam (fine-silty, mixed, mesic, Typic Hapludoll). There was no subsurface drainage tile in the site. The entire experimental area had been planted to maize in the previous 3 years, with 2 exceptions. The east 50 m of the experimental area was planted to oats in 2007, and the south half of the experimental area had strips (north-south orientation) of oats, wheat, and maize followed by red clover and cereal rye cover crops planted on it in 2008. To minimize any carry over effects, our plots were oriented at a 90° angle to these strips (east-west orientation) and the data from the 2009 cropping season, although collected and analyzed, was not included in the final soil NO_3_-N and economic analyses. The site was divided into four blocks (one replicate per block), each containing ten plots. Plots were 18.3 m wide and 190 m long (0.35 ha); large enough to accommodate conventional farm equipment. Crops were harvested from the entire plot area. Soil from each plot was tested annually, and P, K, micronutrients, and lime were applied when necessary to maintain appropriate conditions for crop growth.

### Ethics statement

Part of the experimental site was owned by the City of Sioux Center and part was privately owned. The entire site had been farmed by AJS Farms for many years. Permission to conduct the study was obtained from the City of Sioux Center and from Matt and Leon Schuiteman of AJS farms. No protected species were present on the property. No livestock or other animals were involved in the experiment.

### Cropping systems

We evaluated five different cropping systems during 2009 to 2013 growing seasons (Table 1). Detailed crop management information including crop varieties, seeding rates, herbicides applied, and equipment used are shown in the S6 and S7 Files. All crops for each cropping system were planted each year. Cropping system three, for example, included one plot with oat, another with alfalfa, and another with maize (3a, 3b, 3c) each year. This protocol enabled us to compare cropping systems within and between years.

**Table 1:** Summary of Cropping Systems, Crops Within Cropping Systems, and Nitrogen Fertilizer Treatments.

Cropping Systems (number and name)	Main Crop / Cover Crop Within Each Cropping System ^a^	Nitrogen Fertilizer ^b^
1 –Continuous Maize/Cereal Rye	1—Maize (*Zea mays* L.)/Cereal Rye (*Secale cereale* L.)	25.8 kg N ha^-1^ in mid-April; side-dress application as indicated by the late spring nitrate test ^c^
2—Perennial Grass	2—Smooth Brome Grass (*Bromus inermis* Leyss) and Orchard Grass (*Dactylis glomerata* L.)	None
3 –Oat-Alfalfa-Maize	3a—Oat (*Avena sativa* L.)/Alfalfa (*Medicago sativa* L.) ^d^	None
	3b –Alfalfa (*Medicago sativa* L.)	None
	3c –Maize (*Zea mays* L.)	25.8 kg N ha^-1^ in mid-April
4 –Oat/Red Clover-Maize	4a—Oat (*Avena sativa* L.)/Red Clover (*Trifolium pratense* L.)	None
	4b –Maize (*Zea mays* L.)	25.8 kg N ha^-1^ in mid-April; side-dress application as indicated by the late spring nitrate test
5 –Soybean-Winter Wheat-Maize/Cereal Rye	5a—Soybean (*Glycine max* (L.) Merr.)/Winter Wheat (*Triticum aestivum* L.) ^e^	None
	5b—Winter Wheat (*Triticum aestivum* L.)/Red Clover (*Trifolium pratense* L.) ^f^	None
	5c—Maize (*Zea mays* L.)/Cereal Rye (*Secale cereale* L.)	25.8 kg N ha^-1^ in mid-April; side-dress application as indicated by the late spring nitrate test

^a^ Crop varieties and sources for each year are listed in the supporting information (S6 File). Agronomic information for each cropping system is also shown (S7 File).

^b^ To adjust P and K levels, 112.1 kg ha^-1^ of 7.2-24-24 fertilizer was applied to the entire experimental area in November of 2011 and 2012. As a result, all plots received 8.1 kg ha^-1^ N fertilizer in 2011 and 2012.

^c^ Side-dress N was applied as anhydrous ammonia in 2009, urea-ammonium nitrate (28% N) in 2010 and 2011, and as urea in 2012 and 2013.

^d^ In 2009, a transition year, plots that were scheduled to have second year alfalfa in them were planted to red clover. It established quickly and did a good job of simulating a second year alfalfa stand.

^e^ In the spring of 2009, oat was planted in the winter wheat plots because we were unable to plant winter wheat the previous fall. In the fall of 2010 winter wheat did not establish due to seed quality problems, so we planted spring wheat in April of 2011 instead.

^f^ Red clover established successfully in 2010 but not in the other years, primarily due to dry soil conditions in the fall.

System one was continuous maize with a cereal rye cover crop and side-dressed N. This system was included because we wanted to be able to compare well-managed continuous maize with other cropping systems. Plots were strip-tilled [14] to a depth of 10 cm in mid-April and starter N (urea) was applied, maize was planted in 76 cm wide rows in late April or early May, the late spring nitrate test [15] was conducted in early June, N was side-dressed (at rates indicated by the late spring nitrate test) in late June, and maize was harvested in October. As soon as possible after maize harvest, plots were tilled with a disk harrow and cereal rye was seeded with a grain drill and allowed to overwinter. Strip-tilling in the spring killed 20 cm wide strips of cereal rye. A post emergence herbicide application was used at maize planting to kill the remaining cereal rye, which was typically 25 to 40 cm tall. Any surviving rye was killed by a second post emergence herbicide application in late May or early June.

The second cropping system we investigated was a perennial grass mixture harvested for hay. This system was included because a mixture of perennial grasses is commonly planted around municipal well fields, and it could serve as a benchmark cropping system. Perennial grass in this system was composed of a mix of smooth brome grass (*Bromus inermis* Leyss) and orchard grass (*Dactylis glomerata* L.) and received minimal N fertilizer (Table 1). It was planted in the spring of 2009 and healthy stands persisted for the duration of the experiment. Perennial grass was harvested for hay twice a year during years with normal precipitation (2009, 2010, and 2013), and once per year during very dry years (2011 and 2012).

The third system we evaluated was oat under-seeded with alfalfa, then a year of alfalfa, followed by a year of maize. The oat crop was planted in April and harvested for hay or grain in mid-July or early August, respectively. Alfalfa was seeded at the same time as oat (under-seeded). It grew slowly while competing with the oat crop and was not harvested during the establishment year. The following year alfalfa was harvested three or four times, depending on rainfall and subsequent growth. Spring strip tillage, as described for the continuous maize cropping system, was used to prepare the soil for planting maize and to apply starter N fertilizer. Maize was planted in the tilled strips into a living stand of alfalfa. An herbicide application at planting, and again about 4 weeks later, was used to kill the alfalfa and suppress weeds. Maize had excellent tolerance for the herbicide used, and its growth was unaffected by the applications. Nitrogen fertilizer was not side-dressed in this cropping system, except in 2010 when maize was planted in plots that had not had a full year of alfalfa growth.

The fourth system was one year of oat under-seeded with red clover followed by one year of maize. Oat and red clover were seeded simultaneously in early spring and the oat crop was harvested for either forage or grain in mid-July or early August, respectively. When the oat crop was harvested red clover plants were still quite small and had little effect on oat grain or forage yield. Red clover was allowed to grow through the fall, overwinter, and continue its growth in the spring. Strip tillage in April was used to prepare 20 cm wide strips for planting and to apply starter N fertilizer. Maize was planted on 76 cm rows into the living red clover cover crop (in the tilled strips). Herbicide was applied at planting and about four weeks later to kill the red clover and suppress weeds. Maize tolerance for the herbicide was excellent, and maize growth appeared to be unaffected by the applications. The late spring nitrate test [14] was used to determine side-dressed N rates, N was side-dressed in late June at the rate indicated by the late spring nitrate test, and maize was harvested in October.

The final system was a three year rotation of soybean, winter wheat followed by a red clover cover crop, and maize followed by a cereal rye cover crop. Strip tillage was used to prepare 20 cm wide strips in early May, and soybeans were planted into the strips in 76 cm wide rows in mid-May. Herbicide applications at planting and about four weeks later were used to kill the cereal rye cover crop and control weedy plant species. Soybeans were harvested in October. N fertilizer was not applied to the soybeans. Winter wheat was planted in early to mid- October following soybean harvest. The winter wheat was harvested for grain in early August, and then a red clover cover crop was planted using a broadcast spreader. Red clover establishment was successful in 2010, but not in other years due to dry soil conditions. Strip tillage the following spring was used to prepare 20 cm wide strips for maize planting and to apply starter N fertilizer. Maize was planted on 76 cm rows into the living red clover cover crop (in the tilled strips). Herbicide was applied at planting and about four weeks later to kill the red clover and suppress weeds. The late spring nitrate test [15] was used to determine side-dressed N rates, and maize was harvested in October. As soon as possible after maize harvest plots were tilled with a disk harrow and cereal rye was seeded with a grain drill and allowed to overwinter.

### Data collection

Soil cores (1.9 cm diameter) were taken annually to a depth of 30 cm from each plot in early to mid-June following the protocol for the late spring nitrate test developed by Blackmer [15]. The soil cores (24 per plot) were composited, refrigerated, and then shipped to Midwest Labs in Omaha, NE where they were dried and a standard soil analysis and a NO_3_-N test were completed [16]. The soil NO_3_-N test (to a depth of 30 cm) was repeated in late July to early August. Fall maize stalk NO_3_-N concentrations were determined at the end of the growing season for all maize plots following established protocols [17]. Stalk sampling was done one to three weeks after black layer formation (on the corn kernels) by hand harvesting 15 corn stalk segments per plot. Each segment was 20 cm long, with the lower cut made 15 cm above the soil. Field operations and inputs, and forage and grain yields were recorded annually for each plot. Because we were using commercial farm equipment, the entire plot area was harvested for each crop. A conventional combine was used for grain harvest, while forage crops were mowed, raked, and then baled.

In late October and November of each year, 18 soil cores 4 cm in diameter were collected from each plot with a hydraulic probe. Samples were taken to a depth of 180 cm and divided into 30 cm increments for analysis of residual (at the end of the growing season) NO_3_-N content. For every plot and 30 cm soil increment, the 18 soil cores were composited. Composited samples from all plots and soil depth increments were refrigerated before shipment to Midwest Labs, where they were dried and analyzed. Midwest Labs determined the NO_3_-N concentration in each soil sample and reported it in mg kg^-1^. In November of 2016 we took 20 soil cores 4 cm in diameter and 180 cm deep from the east 2/3 of the plot area, and another 20 samples from the west 1/3 of the plot area. Samples were divided into 30 cm increments, composited, and shipped to Midwest Labs for bulk density analysis. Results from both plot areas were very similar. Mean bulk density for each soil depth (1.19, 1.13, 1.18, 1.19, 1.19, and 1.15 Mg m^-3^, respectively for 0 to 30 cm to 150 to 180 cm increments) was used to calculate soil NO3-N content (kg ha^-1^). The resulting data was used to construct a NO_3_-N content profile (180 cm deep in 30 cm increments) for the soil in each plot and to document the impact of crop rotations and weather on the profile over time [18].

### Economic analyses

An enterprise analysis was conducted for each cropping system for the 2010 to 2013 cropping seasons. Actual costs were used for seed, herbicides, N fertilizer and other direct inputs. Yields of grain and forage were adjusted for moisture content. We used a standard moisture content of 150 g kg^-1^ for maize, 130 g kg^-1^ for soybean, 140 g kg^-1^ for oat, 135 g kg^-1^ for wheat grain, 100 g kg^-1^ for straw, and 150 g kg^-1^ for alfalfa and perennial grass. P and K removal rates were calculated for each crop based on actual yields, and an economic cost for nutrient replacement was assessed to each crop in each year. An opportunity cost was charged for money that was tied up in variable expenses related to the enterprise. Costs for field operations such as fertilizer applications, planting, tillage, and harvesting were assigned based on the Iowa Farm Custom Rate Survey [19]. These rates include the full costs of owning and operating equipment (depreciation, interest, taxes, insurance, repairs, labor, fuel and other costs) and are shown in the S9 File. Land rental rates (S9 File) for each year were based on the results of land rental surveys for Northwest Iowa published by Iowa State University [20]. Annual revenue for each crop was determined by multiplying crop yield by the suggested closing inventory price published annually by Iowa State University for that crop. We examined three different yield scenarios. The first used actual yields from our plots for all crops. The second used actual yields from our plots for all crops except maize and soybean in 2013, for which Sioux County average yields were substituted. The third used average yields from the state of Iowa for all crops and years. United States Department of Agriculture federal farm program direct payments were included in our analysis and were assumed to be $49.40 ha^-1^ yr^-1^ for 2010–2012. Due to farm program changes there was no direct payment for 2013. Profitability (economic profit) for each cropping system was determined by subtracting expenses from total revenue.

### Statistical analyses

A randomized complete block design with four replications was used for the experiment. Each crop phase of each rotation system was present every year so we could accurately assess cropping system effects (Table 1). Each year, cropping system three, for example, included one plot with oat, another with alfalfa, and another with maize (3a, 3b, 3c). For each variable, data from all of the plots in each cropping system was used to calculate a mean value for the cropping system within a given block.

An analysis of variance was conducted for the data from each year using Statistix [21]. Multiple years were analyzed using the procedures for a split-plot design with years as main plots and cropping systems as sub-plots [22]. Interaction terms (with year) were explored, and if appropriate the data was presented by year. NO_3_-N concentration (Mg m^-3^) was analyzed using the procedures for a split-split-plot design with years as main plots, cropping systems as sub-plots, and position in the soil profile (soil depth increments) as sub-sub-plots. Repeated measures of NO_3_-N content within a cropping year were analyzed using the nlme, lme4 and lmerTest packages in the R Project for Statistical Computing [23] software. For the repeated measure analyses, cropping system, crops within cropping systems, and years were treated as fixed effects, replication was considered a random effect, and date of measurement was used as the repeated measure variable. An ANOVA table for the repeated measures analyses is shown in the supporting information (S5 File). Mean separation was accomplished via calculation of LSD values. Means are only shown as significant when ANOVA indicated p values less than 0.05 for the variable being tested.

## Results and discussion

### Soil nitrate

**Fig 1:**
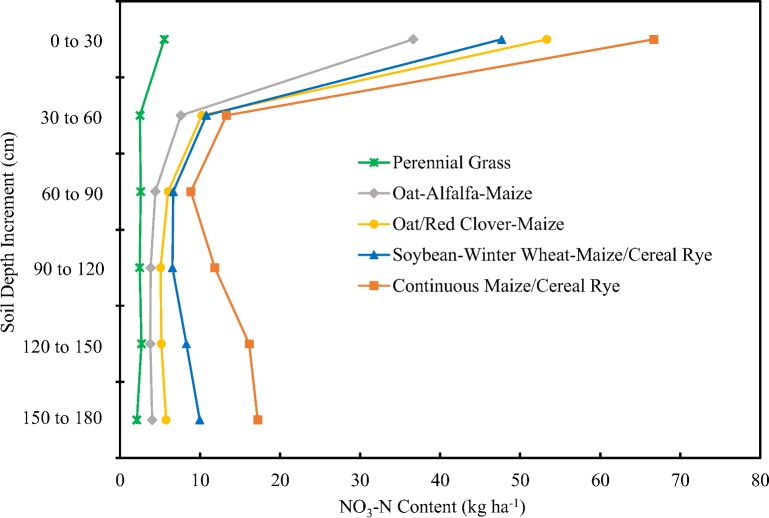
Cropping Systems and Residual Soil NO_3_-N Content. Residual (fall) soil NO_3_-N content as influenced by cropping system and soil depth (30 cm increments to a depth of 180 cm). Data points represent means of the 2010 to 2013 cropping years. See supporting information (S2 File) for mean comparisons.

Cropping systems had a large impact on residual (end of growing season) NO_3_-N content in the upper 180 cm of the soil profile (Fig 1). Continuous maize/cereal rye had higher mean residual NO_3_-N contents than the other cropping systems at all soil depth increments (S2 File), even though multiple management practices were utilized to try and limit residual NO_3_-N content. Rye cover crops following maize production reduce nitrate losses compared to maize grown without cover crops [24–28] and side-dressing N fertilizer based on a late spring nitrate test [15] can reduce N losses compared to fall or spring applications [29,30]. The perennial grass cropping system had the lowest mean NO_3_-N content at all soil depth increments. This was expected, as perennial grass has been shown to reduce the nitrate content of soils and minimize the potential for nitrate leaching [18]. Researchers have reported nitrate losses 30 to 50 times lower from subsurface drainage of plots in perennial grasses compared to plots in continuous maize or a maize-soybean rotation [10]. In the oat-alfalfa-maize system mean residual soil NO_3_-N content was moderate near the soil surface, but nearly as low as under perennial grass at the 90 to 120 cm depth increment and below. This was not surprising, as alfalfa has the ability to remove large quantities of NO_3_-N from agricultural soils [10]. It is a perennial with deep roots, a large N requirement, and long periods of N and water uptake due to early spring and late fall growth. As a result, N removal rates have been found to be two to four times higher with alfalfa than with annual crops like maize [31]. In addition, minimal N fertilizer was applied to these plots (Table 1), as previous research indicates that alfalfa can meet the full N requirement of maize when grown in a three year crop rotation of oat, alfalfa, maize [32]. The oat/red clover-maize system resulted in lower mean residual soil NO_3_-N content than continuous maize. NO_3_-N losses can be reduced by the inclusion of cover crops like red clover in a rotation [27,30]. In addition, legume cover crops planted prior to maize have been shown to reduce soil NO_3_-N concentrations within 5- to 15-cm of the soil surface, most likely due to rapid N mineralization by the legume cover crop, followed by increased N uptake by the maize [33]. The soybean-winter wheat-maize/cereal rye system produced mean residual soil NO_3_-N contents similar to the oat/red clover-maize system. Winter wheat is capable of taking up N made available via mineralization during late fall and early spring, and cereal rye following maize has been shown to effectively reduce soil NO_3_-Nconcentrations [33]. Winter wheat and soybean both received minimal N fertilizer inputs (Table 1).

Cropping systems that included perennial crops (perennial grass, oat-alfalfa-maize, oat/red clover-maize) did not show an increase in mean NO_3_-N concentration from the intermediate depth increment (60 to 90 cm) to the deepest depth increment (150 to 180 cm), while those that included only annual crops (continuous maize/cereal rye and soybean-winter wheat-maize/cereal rye) showed clear increases (S8 File). Our results for the continuous maize cropping system are very similar to those reported for continuous maize in Minnesota [18]. Other researchers examining NO_3_-N movement through the soil profile in field and simulated rainfall conditions have also reported higher NO_3_-N concentrations in the surface and deeper segments of the soil profile than in intermediate depths under some conditions [34,35].

On average, the continuous maize/cereal rye system had a total of 134 kg ha^-1^ of NO_3_-N in the 0 to 180 cm increment of the soil profile, while the perennial grass system had only 18 kg ha^-1^ (Table 2). The other cropping systems had intermediate NO_3_-N contents, with the oat-alfalfa-maize system having a significantly lower NO_3_-N content (60 kg ha^-1^) than the oat/red clover-maize (85 kg ha^-1^) or the soybean-winter wheat-maize/cereal rye (90 kg ha^-1^).

**Table 2:** Total Residual Soil NO_3_-N Content as Affected by Year, Cropping System, and Soil Depth.

Cropping Systems	Residual Soil NO_3_-N Content (kg ha^-1^)^a^									
	0 to 90 cm soil depth increment					90 to 180 cm soil depth increment				
	2010	2011	2012	2013	Mean	2010	2011	2012	2013	Mean
1 –Continuous Maize/Cereal Rye	53.5 a	60.6 a	156.0 a	85.3 a	**88.8 a**	59.2 a	22.2 a	29.4 a	69.9 a	**45.2 a**
2—Perennial Grass	8.3 d	7.8 d	13.7 d	12.6 c	**10.6 d**	5.6 c	6.4 b	6.3 c	10.6 d	**7.2 d**
3 –Oat-Alfalfa-Maize	19.8 c	25.4 c	94.4 c	55.1 b	**48.7 c**	12.9 bc	8.5 b	9.9 c	15.1 d	**11.6 cd**
4 –Oat/Red Clover-Maize	37.1 b	37.3 bc	137.2 ab	66.5 ab	**69.5 b**	18.3 bc	9.0 b	10.8 bc	25.7 c	**15.9 c**
5 –Soybean-Winter Wheat-Maize/Cereal Rye	36.8 b	40.3 b	128.9 b	54.3 b	**65.1 b**	25.9 b	13.2 b	17.2 b	42.6 b	**24.7 b**

Within each soil depth increment and year (vertical columns), differences between cropping systems are denoted by differing lower case letters (LSD, alpha = 0.05).

^a^ Soil NO_3_-N content for the 0 to 90 cm depth increment, and the 90 to 180 cm depth increment were determined by summing the values from the respective 30 cm increments of the soil profile.

Mean NO_3_-N contents in the 0 to 90 cm increment of the soil profile were higher than in the 90 to 180 cm increment for all cropping systems (Table 2, S2 File). The continuous maize/cereal rye cropping system resulted in much higher residual NO_3_-N content in the 0 to 90 cm increment of the soil profile (88.8 kg ha^-1^) than the other cropping systems. Perennial grass had the lowest residual NO_3_-N content in the 0 to 90 cm increment of the soil profile (10.6 kg ha^-1^), followed by the oat-alfalfa-maize system (48.7 kg ha^-1^). Residual NO_3_-N content in the 0 to 90 cm increment was similar for oat/red clover-maize (69.5 kg ha^-1^) and soybean-winter wheat-maize/cereal rye (65.1 kg ha^-1^) systems.

While NO_3_-N throughout the entire soil profile is potentially mobile, the NO_3_-N in the deeper portion is closer to the water table and more difficult for plant roots to access, making it more likely to leach into groundwater and/or into subsurface drainage tile. The continuous maize system had the highest residual NO_3_-N content (45.2 kg ha^-1^) in the 90 to 180 cm increment of the soil profile, while the perennial grass system had the lowest (7.2 kg ha^-1^). However, the residual NO_3_-N content for the oat-alfalfa-maize system between 90 and 180 cm (11.6 kg ha^-1^) was not significantly higher than for perennial grass. The oat/red clover-maize system had a significantly higher NO_3_-N content (15.0 kg ha^-1^) than perennial grass in the 90 to 180 cm depth increment, but was not different than the oat-alfalfa-maize system. The NO_3_-N content in the 90 to 180 cm increment for the soybean-winter wheat-maize system (29.5 kg ha^-1^) was significantly lower than for maize, but higher than for the other cropping systems. This may have been due in part to the inclusion of soybean in this system, as other researchers have suggested that soybean may contribute to NO_3_-N export from agricultural watersheds [36].

An examination of NO_3_-N content in the top 30 cm of soil throughout the growing season provides additional insights into the dynamics of NO_3_-N behavior (Table 3). At each sampling date, soil NO_3_-N content for the continuous maize cropping system was significantly higher than for the other cropping systems. However, the difference was not as large in August, when the crop was growing rapidly, as it was in June and November. In comparison, the perennial grass system had a soil NO_3_-N content that was significantly lower than the other cropping systems in June, August, and November.

**Table 3:** Surface Soil NO_3_-N Content as Affected by Sampling Month, Year, and Cropping System.

		Soil NO_3_-N Content, 0 to 30 cm Soil Depth Increment (kg ha^-1^)				
Sampling Month	Year	Continuous Maize/Cereal Rye	Perennial Grass	Oat-Alfalfa- Maize	Oat/Red Clover—Maize	Soybean- Winter Wheat- Maize/Cereal Rye
June	2010	63.3 a	10.5 c	40.7 b	40.0 b	42.8 b
	2011	38.4 a	6.4 c	27.5 b	23.6 b	25.1 b
	2012	63.0 a	5.1 d	25.5 c	28.4 c	36.3 b
	2013	46.7 a	6.3 c	37.2 b	34.0 b	28.9 b
	**Mean**	**52.9 a**	**7.1 c**	**32.7 b**	**31.5 b**	**33.3 b**
August	2010	43.8 a	11.9 d	23.0 c	36.5 ab	29.0 bc
	2011	28.4 a	6.2 c	8.7 c	18.2 b	18.6 b
	2012	27.5 a	6.1 b	25.2 a	26.2 a	26.4 a
	2013	52.1 a	9.1 b	41.3 a	45.1 a	44.7 a
	**Mean**	**38.0 a**	**8.3 d**	**24.5 c**	**31.5 b**	**29.6 b**
November	2010	30.5 a	3.9 d	11.6 c	19.1 b	20.6 b
	2011	51.5 a	3.8 d	17.9 c	29.2 b	30.8 b
	2012	126.1 a	9.2 d	81.1 c	119.7 ab	106.7 b
	2013	58.6 a	5.1 c	32.6 b	45.2 ab	32.6 b
	**Mean**	**66.7 a**	**5.5 d**	**36.6 c**	**53.3 b**	**47.7 b**

Within each sampling month and year (horizontal rows), differences between cropping systems are denoted by differing lower case letters (LSD, alpha = 0.05).

The comparatively high NO_3_-N content in the soil profile following maize in this experiment could have been due to the application of excess N fertilizer to maize, or to the cropping systems that maize was a component of. Table 4 shows the N fertilizer application rates for all crops in all years. The highest average N fertilizer application rate was for continuous maize (126.4 kg ha^-1^). This rate was based on the late spring nitrate test [15] and is substantially lower than the 213 kg ha^-1^ recommended by Iowa State University for continuous maize [37]. The fall stalk nitrate test [17] was used to assess N availability to the maize crop during the growing season (Table 5). Optimal stalk NO_3_-N concentrations, indicating that the maize plants had adequate N availability during the growing season, are between 700 and 2000 ppm. The highest average stalk nitrate value we observed was 1220 ppm, and the means for the maize crops were between 441 and 482 ppm. This data indicates that N availability, and our N fertilizer rates, were on the conservative (low) end of the spectrum.

**Table 4:** Nitrogen Fertilizer Application Rate and Production Expenses for Each Crop and Cropping System During 2010 to 2013.

Cropping Systems and Crops	N Fertilizer (kg ha^-1^)					Production Expenses ($ ha^-1^)				
	2010	2011	2012	2013	Mean	2010	2011	2012	2013	Mean
**1—Continuous Maize/Cereal Rye**	107.1	151.3	105.6	141.5	**126.4**	1610	1672	1887	1872	**1760**
**2 –Perennial Grass**	0	0	8.1	8.1	**4.1**	879	847	904	1035	**916**
**3 –Oat-Alfalfa-Maize**										
3a –Oat	0	0	8.1	8.1	4.1	776	882	1032	1134	956
3b –Alfalfa	0	0	8.1	8.1	4.1	1354	1195	1242	1452	1311
3c –Maize	25.8	25.8	33.9	33.9	29.85	1425	1376	1465	1665	1483
System Mean	8.6	8.6	16.7	16.7	**12.7**	1185	1151	1247	1417	**1250**
**4 –Oat/Red Clover-Maize**										
4a –Oat/Red Clover	0	0	8.1	8.1	4.1	869	934	1107	1005	979
4b –Maize	132.3	162.6	159.4	96.7	137.8	1833	1620	1709	1655	1704
System Mean	66.2	81.3	83.8	52.4	**70.9**	1351	1242	1408	1330	**1342**
**5 –Soybean-Winter Wheat-Maize/Cereal Rye**										
5a –Soybean	0	0	8.1	8.1	4.1	1074	1242	1378	1346	1260
5b –Winter Wheat	0	0	8.1	8.1	4.1	689	865	988	1035	894
5c –Maize/ Cereal Rye	98.6	178.2	125.8	107.9	127.6	1578	1623	1862	1912	1744
System Mean	32.9	59.4	47.3	41.4	**45.3**	1114	1243	1410	1431	**1299**

**Table 5:** Maize Stalk NO_3_-N Content (ppm) at the End of the Growing Season as Affected by Cropping System and Year.

Cropping System	2010	2011	2012	2013	Mean
**1—Continuous Maize/Cereal Rye**	256	74	1219	354	482 a
**3 –Oat-Alfalfa-Maize**	887	63	140	673	478 a
**4 –Oat/Red Clover-Maize**	1220	55	378	262	475 a
**5 –Soybean-Winter Wheat-Maize/Cereal Rye**	646	107	869	305	441 a
**Mean**	752 a	75 c	651 a	398 b	

Differences among cropping systems and among years are denoted by differing lower case letters (LSD, alpha = 0.05).

**Fig 2:**
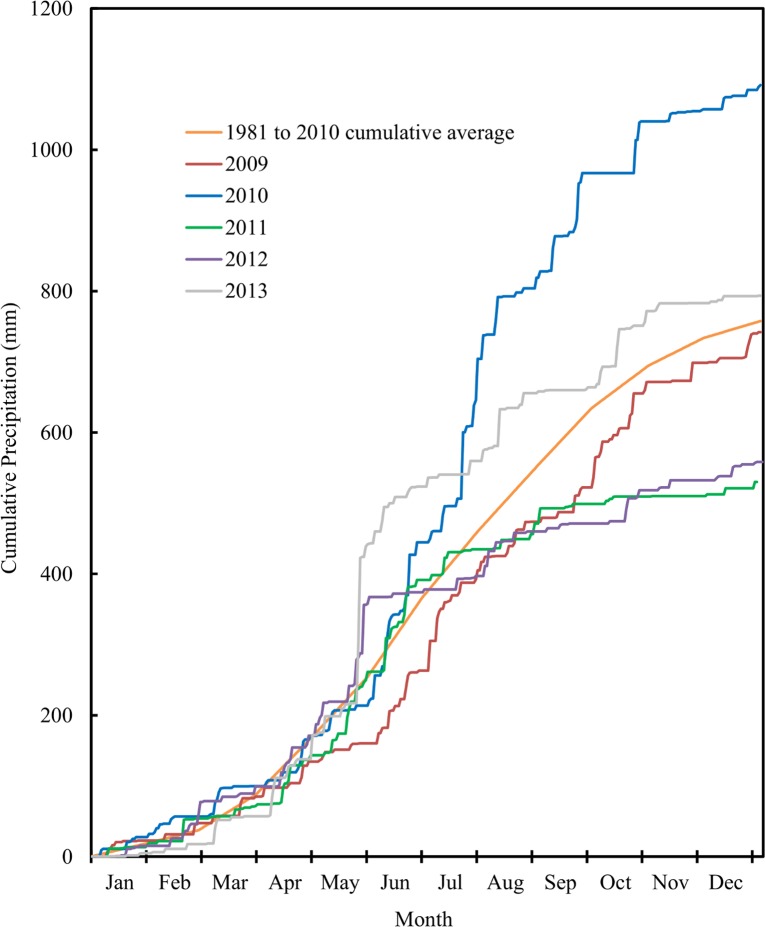
Precipitation for 2009 to 2013. Annual cumulative precipitation for Sioux Center, Iowa during 2009 to 2013, plus the 1981 to 2010 cumulative average.

**Fig 3:**
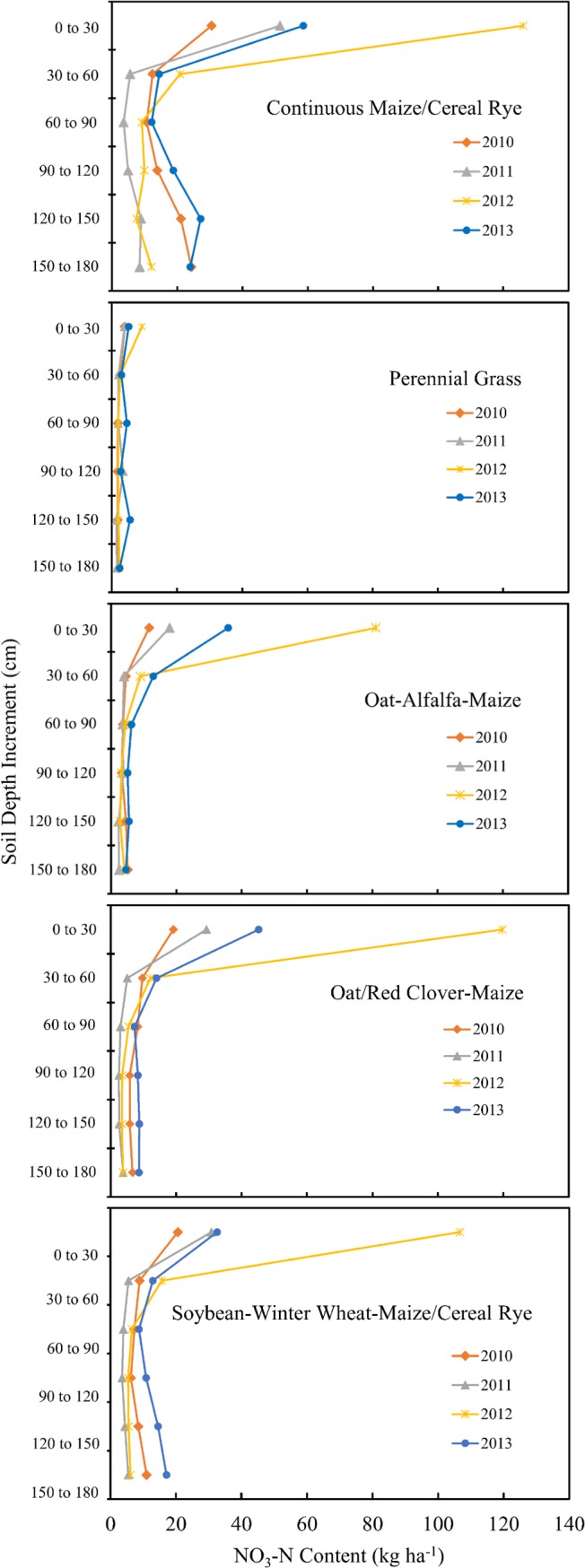
Effect of Year and Cropping System on Residual Soil NO_3_-N Content. Mean residual (fall) soil NO_3_-N content for continuous maize/cereal rye, perennial grass, oat-alfalfa-maize, oat/red clover maize, and soybean-winter wheat-maize/cereal rye cropping systems, as influenced by soil depth increment and year (2010 to 2013).

On an annual basis, soil NO_3_-N content was heavily influenced by precipitation. The 2011 and 2012 cropping seasons were both unusually dry (Fig 2). The lack of moisture inhibited crop growth and reduced N uptake, particularly in the very dry upper portion of the soil profile where N fertilizer had been applied and N had been released by the decomposition of soil organic matter. This resulted in an accumulation of NO_3_-N in the top 60 cm of the soil profile under all cropping systems (Fig 3, Table 2). This effect was seen in the fall of 2012 throughout Iowa [38]. In our plots, high residual NO_3_-N contents in the upper levels of the soil profile in the fall of 2012 apparently resulted in elevated contents in the lower levels of the soil profile in the fall of 2013 in the continuous maize plots and in the soybean-winter wheat-maize/cereal rye cropping system (Fig 3, Table 2). Similar increases in NO3-N contents in the lower levels of the soil profiles were seen in 2010, another relatively wet year. Large changes in nitrate concentrations in the upper and lower portions of soil profile from one year to the next have been documented by others [29], and clearly illustrate the dynamic nature of soil nitrate. Overall, based on the soil NO_3_-N contents observed during this five year study, dry years tended to reduce crop N uptake and yields, which contributed to an accumulation of NO_3_-N in the upper part of the soil profile. When a dry year was followed by a wet year, NO_3_-N tended to be flushed down to deeper levels of the soil profile. This effect was particularly apparent in plots seeded to annual crops. For soil NO_3_-N content, therefore, the cropping system by year interaction was significant for many of the soil depth increments (Fig 3, Table 2) These results indicate that successful management of soil nitrate will depend on the implementation of management strategies that are effective under a wide range of soil moisture conditions.

### Profitability

Production expenses increased gradually from 2010 to 2013 (Table 4). This increase was primarily due to rising land rental rates (S9 File). Rental rates ranged from $464 per ha in 2010 to $699 per ha in 2013. Production expenses were highest for the continuous maize cropping system ($1760 ha^-1^) and lowest for the perennial grass cropping system ($916 ha^-1^). Average expenses for the other three cropping systems were similar ($1250 to $1342 ha^-1^) (Table 4).

Yields of soybean and oat were fairly stable during the 2010 to 2013 cropping seasons, while those of maize, perennial grass, alfalfa and winter wheat were more variable (Table 6). Dry conditions during 2011 (Fig 2) resulted in a substantial reduction in maize yields, and continued lack of rainfall during the 2012 cropping season had a particularly large negative impact on perennial grass production. Year to year variation in the yields of other crops were due to a combination of climate and management factors.

**Table 6:** Crop Yields and Market Prices for Each Crop and Cropping System During 2010 to 2013.

Cropping Systems and Crops	Yield (Mg ha^-1^)					Market Prices^a^ ($ Mg^-1^)				
	2010	2011	2012	2013	Mean	2010	2011	2012	2013	Mean
**1—Continuous Maize/Cereal Rye**	11.72	6.91	10.96	8.75	9.59	187	224	291	171	218
**2 –Perennial Grass**	6.80	3.55	1.27	3.66	3.82	88	110	198	198	149
**3 –Oat-Alfalfa-Maize**										
3a –Oat (grain)	2.79	3.00			2.90	158	234			196
3a –Oat (straw)	2.09	1.50			1.80	77	132			105
3a –Oat (forage)			4.70	4.69	4.70			198	198	198
3b –Alfalfa	11.58	4.54	6.28	10.18	8.15	132	165	248	248	198
3c –Maize	11.90	6.37	8.91	8.99	9.04	187	224	291	171	218
**4 –Oat/Red Clover-Maize**										
4a –Oat/Red Clover (grain)	3.09	4.15			3.62	158	234			196
4a –Oat/Red Clover (straw)	2.09	1.90			2.00	77	132			105
4a –Oat/Red Clover (forage)			4.22	4.99	4.61			198	198	198
4b –Maize	12.18	6.95	8.04	4.91	8.02	187	224	291	171	218
**5 –Soybean-Winter Wheat-Maize/Cereal Rye**										
5a –Soybean	4.84	2.44	3.38	2.53	3.30	426	391	486	472	444
5b –Winter Wheat (grain)	2.03	1.52	3.46	3.07	2.52	211	226	308	229	244
5b –Winter Wheat (straw)	1.32	1.16	1.54	1.45	1.37	77	132	110	110	107
5c –Maize/Cereal Rye	12.53	7.79	11.49	7.32	9.79	187	224	291	171	218

^a^ Market prices are Iowa State University suggested closing inventory prices for each crop in each year.

Market prices varied greatly from one year to the next (Table 6). For example, the market price for maize was $291 Mg^-1^ in 2012, but only $171 Mg^-1^ in 2013. Perennial grass hay was valued at $88 Mg^-1^ in 2010, and increased to $198 Mg^-1^ in 2012. Oat and winter wheat straw were valued at $77 Mg^-1^ in 2010 and $132 Mg^-1^ in 2011.

Profitability of each cropping system was estimated using three different procedures (Table 7). This was done to provide as broad of an assessment as possible, given the large fluctuations in yield and price during the years the experiment was conducted. Surprisingly, all three methods resulted in fairly similar outcomes. Utilizing adjusted means from our experiment, continuous maize/cereal rye was the most profitable cropping system ($531 ha^-1^) and perennial grass was the least profitable ($-384 ha^-1^). However, perennial grass was primarily grown as a control treatment and therefore did not receive N fertilizer. Appropriate fertilization would likely have increased yields with little or no increase in soil nitrate concentrations [10,18,39]. The oat-alfalfa-maize system generated an average profit of $264 ha^-1^, the oat/red clover-maize system $140 ha^-1^ and the soybean-winter wheat-maize/cereal rye $347 ha^-1^.

**Table 7:** Profitability of Cropping Systems.

Cropping Systems and Crops	Net Profit ($ Ha^-1^)						
	Experimental Data^a^					Adjusted Means^b^	Iowa Averages^c^
	2010	2011	2012	2013	Mean	2010 to 2013	2010 to 2013
**1—Continuous Maize/Cereal Rye**	633	-74	1343	-369	**383**	**531**	**435**
**2 –Perennial Grass**	-231	-408	-596	-300	**-384**	**-384**	**-193**
**3 –Oat-Alfalfa-Maize**	318	-155	459	251	**218**	**264**	**212**
**4 –Oat/Red Clover-Maize**	161	-45	230	-410	**-16**	**140**	**103**
**5 –Soybean-Winter Wheat-Maize/Cereal Rye**	582	-130	722	-317	**214**	**347**	**291**

^a^ Profitability is based on revenue from plot yields at market prices (Iowa State University suggested closing inventory prices from each crop in each year) from 2010 through 2013 [40,41], less actual costs.

^b^ In 2013, Sioux County average yields for maize (12.18 Mg ha^-1^) and soybean (4.11 Mg ha^-1^) were used [42] instead of actual yields. Actual yields were artificially low due to poor soybean stands and other management challenges.

^c^ Profitability is based on revenue from average annual yields and market prices for the state of Iowa from 2010 through 2013 [40–42], less actual costs.

### The intersection of soil nitrate and profitability

At first glance, low to moderate levels of residual soil NO_3_-N and moderate to high profitability don’t seem to occur simultaneously. The cropping system with the lowest residual soil NO_3_-N levels (perennial grass) was the least profitable, and the system with the highest soil NO_3_-N levels (continuous maize/cereal rye) was the most profitable (Tables 2 and 7). However, a closer look indicates that there are some promising options for cropping systems which are both profitable and have low concentrations of residual NO_3_-N.

Total residual soil NO_3_-N levels in the 90 to 180 cm soil depth increments were nearly as low in the oat-alfalfa-maize and oat/red clover-maize systems as they were in the perennial grass system. These cropping systems, while not as profitable as continuous maize/cereal rye, did generate positive returns and would minimize nitrate leaching. They also point us toward some other alternatives. Results from the oat-alfalfa-maize and oat/red clover-maize cropping systems suggest that incorporating a tap-rooted perennial species into the rotation in at least one out of three years may result in very little NO_3_-N loss in fields without subsurface tile drainage. Given this data, rotations like maize-maize-alfalfa-alfalfa or maize-soybean-winter wheat-alfalfa-alfalfa appear to have potential from both economic and environmental points of view. The latter rotation would require less N fertilizer, and could allow for fall seeding of alfalfa. Seeding alfalfa in the fall would result in living soil cover for more of the year than spring establishment, fewer weed problems, and increased forage yields the first full growing season [43]. Additional research exploring these and other options from both economic and environmental points of view is needed.

### Future directions

Iowa State University Extension has developed a model to evaluate the profitability of Midwestern crop rotations using optimal nitrogen fertilization rates [44]. Currently, this model is limited to variations of a corn and soybean rotation. Expansion of this model (or development of new models) to include additional crops and an estimate of residual NO_3_-N levels could result in a very useful tool for identifying systems which minimize the risk of NO_3_-N leaching while providing positive economic returns. Use of this type of model could allow producers and municipalities to readily evaluate the risk of NO_3_-N losses, and the profitability of a range of cropping systems. This information would facilitate the development of cropping systems tailored to specific situations.

Currently, the crops that most effectively reduce residual soil NO_3_-N levels are perennial forage species. These crops require specialized equipment to produce, involve more management and labor than maize and soybeans, and may be difficult to market in regions with low ruminant livestock populations. Perennial grain-producing crops could avoid some of these constraints. Researchers at the Land Institute in Salina, KS have made good progress breeding intermediate wheatgrass (a perennial) for seed production [45]. This new crop, called ‘Kernza’, has been shown to effectively reduce residual soil NO_3_-N concentrations compared to annual crops [39]. Production of Kernza in alluvial aquifers near shallow municipal wells could become an attractive option in the next 5 to 10 years.

Breeding maize and other annual crops for increased ability to extract soil N at low concentrations may also be feasible [46–48]. Recent developments in the study of nitrogen use efficiency suggest that it may be possible to develop maize cultivars (and perhaps cultivars of other annual crops) for increased ability to extract N from soils with relatively low soil NO_3_-N content [49].

To successfully reduce NO_3_-N leaching from crop fields, agricultural policy needs to reward management practices that re-couple the C and N cycles, and prevent nitrate from moving down through the soil profile [50]. The USDA Conservation Stewardship Program is a step in the right direction [51], as is the Conservation Reserve Program Wellhead Protection program. In sensitive areas of the maize-soybean belt of the United States stronger incentives could be given for planting a crop in rotation that reduces NO_3_-N concentrations in the lower levels of the soil profile. Alternatively, eligibility for federal government farm program benefits in sensitive areas could be dependent on including such a crop in rotation. The removal of nitrogen from the lower soil profile in wellhead capture zones, and on the broader landscape, represents a common good for society and therefore should be promoted by a range of well-designed and effective public policies.

## Supporting information

S1 File USDA Farm Service Agency CRP-WHP Signup in Iowa. (DOCX)

S2 File Residual (Late Fall) Soil NO_3_-N Content as Affected by Cropping System, Crops Within Cropping Systems and Soil Depth (30 cm increments to a depth of 180 cm). Data points represent means of the 2010 to 2013 cropping years.
(DOCX)

S3 File Striking Image–Photo of Experimental Site. (JPG)

S4 File Soil Test, Stalk Nitrate, Yield, and Nitrogen Application Data. (XLSX)

S5 File ANOVA Table for a Repeated Measures Analysis. (DOCX)

S6 File Crop Varieties and Seed Sources. (DOCX)

S7 File Agronomic Information. (DOCX)

S8 File Residual Soil NO_3_-N Concentration as Affected by Soil Depth Increment and Cropping System. (DOCX)

S9 File Field Operations and Input Costs by Year. (DOCX)
