# Supplementary material for: Residual soil nitrate content and profitability of five cropping systems in northwest Iowa
Source: PLoS One. 2017 Mar 1;12(3):e0171994. doi: 10.1371/journal.pone.0171994 (PMC5332022; doi:10.1371/journal.pone.0171994)
Supplement: S1 File — (DOCX) [file pone.0171994.s001.docx]

**From:** Holcombe, Robin - FSA, Urbandale, IA
**Sent:** Tuesday, December 08, 2015 3:14 PM
**To:** 'Robert.DeHaan@dordt.edu' <[Robert.DeHaan@dordt.edu](mailto:Robert.DeHaan@dordt.edu)>
**Cc:** Goettsch, Curt - FSA, Urbandale, IA <[Curt.Goettsch@ia.usda.gov](mailto:Curt.Goettsch@ia.usda.gov)>
**Subject:** Wellhead protection acres.xls

Robb,

Attached are the Wellhead acres for Iowa.

Robin Holcombe

515 331 8441
