# Supplementary material for: Residual soil nitrate content and profitability of five cropping systems in northwest Iowa
Source: PLoS One. 2017 Mar 1;12(3):e0171994. doi: 10.1371/journal.pone.0171994 (PMC5332022; doi:10.1371/journal.pone.0171994)
Supplement: S2 File — Data points represent means of the 2010 to 2013 cropping years. (DOCX) [file pone.0171994.s002.docx]

**S2 File. Residual (Late Fall) Soil NO_3_-N Content as Affected by Cropping System and Soil Depth (30 cm increments to a depth of 180 cm).** Data points represent means of the 2010 to 2013 cropping years.

| **Cropping Systems** | **Residual Soil NO_3_-N Content (kg ha^-1^)** | | | | | |
| --- | --- | --- | --- | --- | --- | --- |
|  | **0 to 30 cm soil depth** | **30 to 60 cm soil depth** | **60 to 90 cm soil depth** | **90 to 120 cm soil depth** | **120 to 150 cm soil depth** | **150 to 180 cm soil depth** |
| 1 – Continuous Maize/Cereal Rye | 66.7 a | 13.3 a | 8.8 a | 11.8 a | 16.2 a | 17.2 a |
| 2 - Perennial Grass | 5.5 d | 2.5 d | 2.6 d | 2.5 d | 2.7 d | 2.1 e |
| 3 – Oat-Alfalfa-Maize | 36.6 c | 7.6 c | 4.4 c | 3.8 cd | 3.8 cd | 4.0 d |
| 4 – Oat/Red Clover-Maize | 53.3 b | 10.2 b | 6.0 b | 5.1 bc | 5.2 c | 5.7 c |
| 5 – Soybean-Winter Wheat-Maize/Cereal Rye | 47.7 b | 10.8 b | 6.6 b | 6.6 b | 8.3 b | 9.9 b |

Within each soil depth increment (vertical columns) differences in NO_3_-N content between cropping systems are denoted by differing lower case letters (LSD, alpha = 0.05). Valid comparisons of NO_3_-N content can only be made within each soil depth increment, not across depth increments.
