# Supplementary figures and images for: Residual soil nitrate content and profitability of five cropping systems in northwest Iowa
Source: PLoS One. 2017 Mar 1;12(3):e0171994. doi: 10.1371/journal.pone.0171994 (PMC5332022; doi:10.1371/journal.pone.0171994)

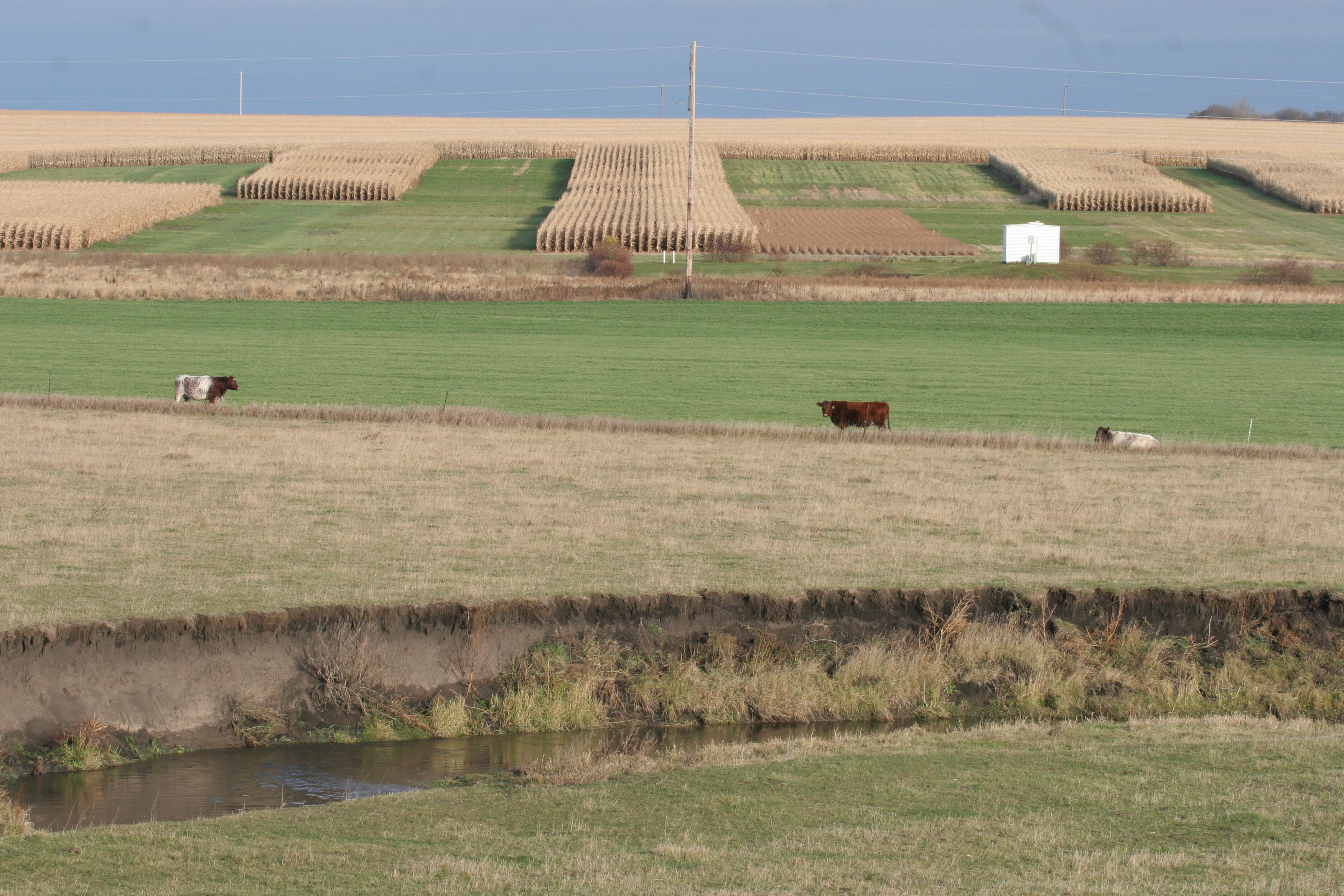

Supplement: S3 File — (JPG) [file pone.0171994.s003.jpg]
