# Supplementary material for: Residual soil nitrate content and profitability of five cropping systems in northwest Iowa
Source: PLoS One. 2017 Mar 1;12(3):e0171994. doi: 10.1371/journal.pone.0171994 (PMC5332022; doi:10.1371/journal.pone.0171994)
Supplement: S5 File — (DOCX) [file pone.0171994.s005.docx]

S5 File. ANOVA Table for a Repeated Measures Analysis.

Results from a repeated measure analysis of NO_3_-N content (kg ha^-1^) in the 0 to 30 cm depth of the soil profile in June, August, and November of 2010 to 2013. Cropping systems include one to three different crops. In systems with two or more crops mean responses were determined and utilized in the analysis. For this analysis cropping systems and years are fixed effect variables, replication is a random effect variable, and sample date is a repeated measure variable.

| Source | Numerator DF | Denominator DF | Sum of Squares | Mean Square | F Value | P Value |
| --- | --- | --- | --- | --- | --- | --- |
| Cropping System | 4 | 177 | 53165 | 13291 | 194.9 | <0.001 |
| Year | 3 | 177 | 20882 | 6961 | 102.1 | <0.001 |
| Sample Date | 2 | 177 | 10084 | 5042 | 73.9 | <0.001 |
| Cropping System x Year | 12 | 177 | 6132 | 511 | 7.5 | <0.001 |
| Cropping System x Sample Date | 8 | 177 | 5789 | 724 | 10.6 | <0.001 |
| Year x Sample Date | 6 | 177 | 48257 | 8043 | 117.9 | <0.001 |
| Cropping System x Year x Sample Date | 24 | 177 | 11936 | 497 | 7.3 | <0.001 |
