# Supplementary material for: Residual soil nitrate content and profitability of five cropping systems in northwest Iowa
Source: PLoS One. 2017 Mar 1;12(3):e0171994. doi: 10.1371/journal.pone.0171994 (PMC5332022; doi:10.1371/journal.pone.0171994)
Supplement: S6 File — (DOCX) [file pone.0171994.s006.docx]

**S6 File. Crop Varieties and Seed Sources.**

| **Common Name** | **Scientific Name** | **Year** | **Variety** | **Source** |
| --- | --- | --- | --- | --- |
| Maize | *Zea mays* L. | 2009 | Pioneer 33W84 Pioneer 35K33^a^ | Purchased |
|  |  | 2010 | Pioneer 37K11 Pioneer 0115XR | Purchased |
|  |  | 2011 | Pioneer 9990XR | Purchased |
|  |  | 2012 | Pioneer 0528AM1 Pioneer 05333XR | Purchased |
|  |  | 2013 | Pioneer 0453AM | Purchased |
| Cereal Rye | *Secale cereale* L. | 2009 | VNS^b^ | Purchased |
|  |  | 2010 | VNS | Purchased |
|  |  | 2011 | VNS | Purchased |
|  |  | 2012 | VNS | Purchased |
|  |  | 2013 | VNS | Purchased |
| Smooth Brome | *Bromus inermis* Leyss | 2009 to 2013 | VNS | Purchased |
| Orchard Grass | *Dactylis glomerata* L. | 2009 to 2013 | VNS | Purchased |
| Oat | *Avena sativa* L. | 2009 | Stallion | Purchased |
|  |  | 2010 | Souris | Purchased |
|  |  | 2011 | VNS (Souris) | Saved^b^ |
|  |  | 2012 | VNS (Souris) | Saved |
|  |  | 2013 | VNS (Souris) | Saved |
| Alfalfa | *Medicago sativa* L. | 2009 | Pioneer 53H92 | Purchased |
|  |  | 2010 | Pioneer 53H92 | Purchased |
|  |  | 2011 | Pioneer 53H92 | Purchased |
|  |  | 2012 | Pioneer 55V12 | Purchased |
|  |  | 2013 | Pioneer 55V12 | Purchased |
| Red Clover | *Trifolium pratense* L. | 2009 | VNS | Purchased |
|  |  | 2010 | VNS | Purchased |
|  |  | 2011 | VNS | Purchased |
|  |  | 2012 | VNS | Purchased |
|  |  | 2013 | VNS | Purchased |
| Soybean | *Glycine max* (L.) Merr. | 2009 | Pioneer 92Y30 | Purchased |
|  |  | 2010 | Pioneer 91Y90 | Purchased |
|  |  | 2011 | Pioneer 91Y90 | Purchased |
|  |  | 2012 | Pioneer 91Y90 | Purchased |
|  |  | 2013 | Pioneer 91Y90 | Purchased |
| Winter Wheat | *Triticum aestivum* L. | 2009 | VNS | Purchased |
|  |  | 2010 | Overland | Purchased |
|  |  | 2011 | Steele | Purchased |
|  |  | 2012 | VNS (Steele) | Saved |
|  |  | 2013 | VNS (Steele) | Saved |

^a^When two varieties are listed, half (12 rows) of every plot was planted to each variety.

^b^Variety not stated.

^c^Seed saved after harvest the previous year and planted in the current year.
