# Supplementary material for: Residual soil nitrate content and profitability of five cropping systems in northwest Iowa
Source: PLoS One. 2017 Mar 1;12(3):e0171994. doi: 10.1371/journal.pone.0171994 (PMC5332022; doi:10.1371/journal.pone.0171994)
Supplement: S7 File — (DOCX) [file pone.0171994.s007.docx]

**S7 File. Agronomic Information.** Plots were not irrigated. In the fall of 2011 and 2012 all plots received a broadcast application of 96 kg ha^-1^ lime (SuperCal 98G Pelletized Lime), and 112.1 kg ha^-1^ of 7.2-24-24 fertilizer.^a^ Crop residue was left on plots. Standard farm equipment was used for plot management.^b^

| **Cropping Systems** | **Main Crop** | **Tillage Method** | **Planting time** | **Planting rate** | **Herbicide** | **Harvest method** | **Harvest time** |
| --- | --- | --- | --- | --- | --- | --- | --- |
| 1 Continuous Maize/cereal rye | Maize | Maize-strip till/planter  cereal rye - disk/drill | Maize - late April  Cereal Rye - October | Maize - 83503 to 88189 seeds ha^-1^  Cereal Rye – 100.8 kg ha^-1^ | *2,4-D^c^*  *Herbimax^d^*  *Glyphosate^e^* | Combine | Fall |
| 2 Perennial Grass | Perennial Grass | Drill/disk (only in 2009) | Late March | Smooth brome 9 kg ha^-1^  Orchard grass 11.2 kg ha^-1^ | None | Baler | Summer |
| 3 Oat-Alfalfa-Maize | 3a Oat | Oat/Alfalfa - disk/drill | Late March | 100.8 kg ha^-1^ | None | Combine for grain  Baler for straw | Summer |
|  | 3b Alfalfa | Alfalfa - drilled with oat the previous year | Late March | 20.2 kg ha^-1^ | None | Baler | Summer |
|  | 3c Maize | Maize-strip till/planter | Late April | 83503 to 88189 seeds ha^-1^ | *2,4-D*  *Herbimax*  *Glyphosate* | Combine | Fall |
| 4 Oat/Red Clover-Maize | 4a Oat | Oat/Red Clover - disk/drill | Late March | Oat - 100.8 kg ha^-1^  Red Clover - 20.2 kg ha^-1^ | None | Combine for grain  Baler for straw | Summer |
|  | 4b Maize | Maize-strip till/planter | Late April | 83503 to 88189 seeds ha^-1^ | *2,4-D*  *Herbimax*  *Glyphosate* | Combine | Fall |
| 5 Soybean-Winter Wheat-Maize/Cereal Rye | 5a Soybean | Soybean-strip till/planter | Early May-soybean | 264320 to 348786 seeds ha^-1^ | *Glyphosate* | Combine | Fall |
|  | 5b Winter Wheat | Winter Wheat-disk/drill after soybean (previous fall)  Red Clover-disk/drill | Winter Wheat - October (previous fall)  Red Clover - August | 100.8 kg ha^-1^  20.2 kg ha^-1^ | None | Combine for grain  Baler for straw | Summer  Summer |
|  | 5c Maize | Maize-strip till/planter  Cereal Rye - disk/drill | Maize - late April  Cereal Rye - October | Maize - 83503 to 88189 seeds ha^-1^  Cereal Rye - 100.8 kg ha^-1^ | *2,4-D*  *Herbimax*  *Glyphosate* | Combine | Fall |

1. Table 4.
2. Tractors: John Deere 9230, 9220, 4430, 4020, Farmall 706. Combines: John Deere 9760 STS and 9770 STS. Round baler: John Deere 566. Square baler: New Holland 67. Planter: John Deere 1770 NT. Strip till machine: Environmental Tillage Systems Warrior. Drill: John Deere 850. Disk: John Deere 637. Sprayer: Fast (60ft boom). Mower: Hesston. Rake: Tonutti.
3. Dimethylamine salt of 2,4-Dichloro-phenoxyacetic acid.
4. Herbimax^R^ is an oil surfactant which increases penetration and activity of post emergence herbicides.
5. Glyphosate as isopropylamine salt.
