# Supplementary material for: Residual soil nitrate content and profitability of five cropping systems in northwest Iowa
Source: PLoS One. 2017 Mar 1;12(3):e0171994. doi: 10.1371/journal.pone.0171994 (PMC5332022; doi:10.1371/journal.pone.0171994)
Supplement: S8 File — (DOCX) [file pone.0171994.s008.docx]

| Soil Depth Increment (cm) | Residual Soil NO_3_-N Concentration (mg kg^-1^) ^a^ | | | | |
| --- | --- | --- | --- | --- | --- |
|  | Continuous Maize/Cereal Rye | Perennial Grass | Oat-Alfalfa-Maize | Oat/Red Clover-Maize | Soybean-Winter Wheat-Maize/ Cereal Rye |
| 0 to 30 | 18.68 | 1.55 | 10.26 | 14.94 | 13.36 |
| 30 to 60 | 3.92 | 0.73 | 2.24 | 3.02 | 3.18 |
| 60 to 90 | 2.50 | 0.73 | 1.25 | 1.70 | 1.87 |
| 90 to 120 | 3.32 | 0.69 | 1.07 | 1.42 | 1.83 |
| 120 to 150 | 4.52 | 0.75 | 1.06 | 1.45 | 2.32 |
| 150 to 180 | 5.00 | 0.61 | 1.16 | 1.66 | 2.89 |

**S8 File. Residual Soil NO_3_-N Concentration as Affected by Soil Depth Increment and Cropping System.** Data points represent means of the 2010 to 2013 cropping years.

^a^ LSD (0.05) is 1.00 for comparison of means within cropping systems (vertical columns) and 1.05 for comparison of means between cropping systems (horizontally) at the same or different soil depths.
