# Supplementary material for: Residual soil nitrate content and profitability of five cropping systems in northwest Iowa
Source: PLoS One. 2017 Mar 1;12(3):e0171994. doi: 10.1371/journal.pone.0171994 (PMC5332022; doi:10.1371/journal.pone.0171994)
Supplement: S9 File — (DOCX) [file pone.0171994.s009.docx]

**S9 File. Cropping System Expenses.**

| **Expense Categories** |  | **Year** | | | |
| --- | --- | --- | --- | --- | --- |
| **Field Operations** | **Expense Units** | **2010** | **2011** | **2012** | **2013** |
| Strip Tillage | $ Ha^-1^ | 28.66 | 29.16 | 31.01 | 33.61 |
| Planting | $ Ha^-1^ | 41.39 | 45.96 | 51.64 | 51.89 |
| Spraying | $ Ha^-1^ | 15.07 | 14.95 | 15.69 | 16.43 |
| Broadcast (granular) Fertilizer Application | $ Ha^-1^ | 18.53 | 17.30 | 19.77 | 24.71 |
| Nitrogen Fertilizer Application - Ammonia | $ Ha^-1^ | 37.44 | 38.42 | 42.25 | 40.03 |
| Mowing Hay | $ Ha^-1^ | 30.64 | 30.64 | 30.64 | 32.12 |
| Raking Hay | $ Ha^-1^ | 13.96 | 14.21 | 15.32 | 15.44 |
| Baling Hay – Large Round | $ Bale^-1^ | 9.80 | 9.45 | 9.40 | 10.45 |
| Baling Hay – Small Square | $ Bale^-1^ | 0.50 | 0.50 | 0.50 | 0.60 |
|  |  |  |  |  |  |
| **Land Rent** | $ Ha^-1^ | 464.55 | 553.50 | 659.76 | 699.29 |
